# Physicochemical characterization and phenolic compound content of flour from blended juice residues

**DOI:** 10.1002/jsfa.70523

**Published:** 2026-02-19

**Authors:** Letícia da Cunha Espinosa, Aldrey Nathália Ribeiro Corrêa, Adriano Brandelli, Simone Hickmann Flôres, Alessandro de Oliveira Rios

**Affiliations:** ^1^ Bioactive Compounds Laboratory Food Science and Technology Institute, Federal University of Rio Grande do Sul Porto Alegre Brazil; ^2^ Laboratory of Nanotechnology and Applied Microbiology Food Science and Technology Institute, Federal University of Rio Grande do Sul Porto Alegre Brazil

**Keywords:** by‐products, dietary fiber, drying process, residue, sensory analysis, sustainability

## Abstract

**BACKGROUND:**

The production of fruit juices generates substantial amounts of residue, including peels, seeds, pomace, stalks, and leaves, which are rich in nutritionally relevant compounds, such as fiber and bioactives. The aims of this study were to evaluate drying conditions for converting mixed juice residue, composed of apple, beetroot, lemon, and ginger, into flour; to characterize its physical, chemical, and functional properties; and to assess its application in food products.

**RESULTS:**

Drying the residue at 70 °C for a shorter time produced flour with superior color retention, higher phenolic compound content, and greater betacyanin concentration than fresh waste and the other flours dried at 50 and 60 °C. Ten phenolic compounds were identified. The flour demonstrated antifungal activity against *Fusarium* species, indicating its potential as a natural preservative ingredient. Partial replacement of wheat flour with residue flour in cake formulation yielded a product with over 80% overall acceptance in sensory analysis and a 40% higher fiber content than the cake without added residue flour.

**CONCLUSIONS:**

The results demonstrate the viability of using agro‐industrial residues as nutritionally valuable food ingredients that are sensorially attractive to consumers. This approach aims to sustainably valorize important food components that are typically discarded. © 2026 The Author(s). *Journal of the Science of Food and Agriculture* published by John Wiley & Sons Ltd on behalf of Society of Chemical Industry.

## INTRODUCTION

Residues from fruit and vegetable juice production are estimated to account for 25% to 65% of the raw material, depending on their source.These residues include peels, seeds, pomace, stalks, and leaves, which are generally used for animal feed or discarded, generating additional costs for industry and contributing to environmental impacts. Market trends and reports from institutions such as Innova Market Insights indicate continuous growth in natural juice consumption, driven by the pursuit of healthy eating habits.^1^ This increased demand is resulting in a proportional rise in residue generation, highlighting the need for sustainable reuse strategies.

Residues from fruit and vegetable processing are important sources of dietary fiber and bioactive compounds and therefore have strong potential for reuse in the development of new ingredients or food products. Their incorporation into traditional products, such as bakery and confectionery items, can increase their nutritional value and provide health benefits to consumers, largely due to the presence of functional components associated with the prevention of non‐communicable chronic diseases. The use of these residues also represents a sustainable strategy for waste reduction and promotes more comprehensive use of food resources.

Dietary fibers are present in a wide variety of plant‐based foods, although their concentration varies depending on the type and part of the plant. In certain vegetables, such as beetroot, fiber content can reach approximately 8% of fresh matter. Fruits such as peeled apples and citrus have a fiber content of around 2%, depending on variety and ripening stage.^2^ However, these values refer to whole foods that contain high levels of moisture. During juice production, a significant portion of the liquid fraction is removed, concentrating insoluble constituents, including fibers, in the solid residue.

This observation is consistent with recent findings. Richards *et al*.^3^ evaluated dehydrated carrot pomace and reported a total dietary fiber content of 518.4 ± 70.8 g kg^−1^, comprising 295.1 ± 26.6 g kg^−1^ insoluble fiber and 223.3 ± 53.0 g kg^−1^ soluble fiber. Similarly, Hotchkiss *et al*.^4^ reported a total fiber content of 508 g kg⁻¹ in beetroot pomace, with insoluble fiber (401 g kg⁻¹) predominating over soluble fiber (107 g kg⁻¹). These results underscore the functional potential of agro‐industrial residues, particularly as sources of dietary fiber. Their valorization represents a promising strategy to enhance dietary fiber intake while promoting the sustainable use of food resources.

Dietary fiber provides numerous health benefits. A simple dietary modification – replacing white bread with fiber‐rich bread – has been shown to improve gut microbiota and increase the population of short‐chain fatty acid‐producing microorganisms.^5^ Based on these results, the authors concluded that increased fiber intake benefits gastrointestinal health and overall metabolism. Other studies have reported positive effects on weight management and cholesterol regulation,^6^ as well as a reduced risk of cardiovascular diseases and cancer.^7^

Phenolic compounds are plant‐derived bioactives with beneficial effects on both plant defense mechanisms and human health. Although not considered essential nutrients, their consumption has been associated with antioxidant, anti‐inflammatory, antimicrobial, anticancer, neuroprotective,^8^ and antidiabetic effects.^9^ Among these compounds, *m*‐coumaric acid exhibits antioxidant and antidiabetic activity, reducing glucose and glycated hemoglobin levels.^10^ Gallic acid, one of the most abundant phenolics, demonstrates antibacterial and anti‐inflammatory effects and supports angiogenesis and epidermal regeneration.^11^ Protocatechuic acid has shown benefits in neurodegenerative conditions, possibly by decreasing inflammatory mediators and oxidative stress markers.^12^

To promote the valorization of agro‐industrial residues rich in bioactive components and support sustainability, this study transformed residues from mixed juice production – comprising apple, beetroot, lemon, and ginger – into a flour‐based ingredient. The resulting flour was characterized in terms of its physicochemical and functional properties, including the identification and quantification of phenolic compounds. Its potential application was assessed by incorporating it into a cake formulation, developed as a fiber‐enriched food product.

## MATERIALS AND METHODS

### Blended juice residue

Mixed juice residues, comprising apple, beetroot, lemon, and ginger, were kindly provided by Super Labs (Eldorado do Sul, Rio Grande do Sul, Brazil) and were processed separately.The material, consisting of peels, seeds, and stalks, was stored at −18 °C until use.

The production of 1 L of mixed juice (55% apple, 33% beetroot, 10% lemon, and 2% ginger) generated approximately 638 g of residue, comprising 37.5% apple, 29% beetroot, 30.5% lemon, and 3% ginger. This corresponded to about 37% of the total fruit weight.

### Blended residue flour

The residues were dried in a forced‐air circulation oven at 50, 60, and 70 °C, following previously reported protocols, until final moisture content of 12% to 15% was reached, in accordance with CODEX Stan 152–1985.^13^ Previous studies have dried pitaya peel at 60 °C,^14^ beetroot at 60–80 °C – with 70 °C identified as optimal for preserving antioxidant activity and betalain content^15^ – and orange peel at 60 °C.^16^ Based on these reports and similarities among plant matrices, these temperatures were selected, with the inclusion of 50 °C to evaluate the effect of lower drying temperature on the preservation of bioactive compounds and color.

Drying curves were constructed for each temperature until constant weight was reached. Samples (5 g) were weighed at 60 min intervals to monitor the drying rate and determine the time required to reach equilibrium moisture under each condition. The resulting data were used to compare drying efficiency across temperatures, and the optimal condition was selected based on color, total phenolic content, and betacyanin concentration.

Particle size separation was performed using sieves (Bertel, Caieiras, SP, Brazil), to classify the flour into two fractions: ≥ 60 mesh (fine flour) and < 60 mesh (coarse flour). The samples were vacuum sealed, protected from light, and stored at 25 °C until analysis.

### Analyses

The resulting flour was analyzed for moisture, pH, ash, lipids, and proteins, following AOAC methods.^17^ Carbohydrate content was estimated by difference. Total, soluble, and insoluble dietary fiber were determined using the enzymatic‐gravimetric method, again following AOAC methods.^17^ Results were expressed as g kg^−1^ dry matter (DM). Water activity (Aw) was measured using a water activity analyzer (AquaLab, model AquaLab PRE, Pullman, WA, USA). All analyses were performed in triplicate.

#### Functional properties

The oil holding capacity (OHC) and water holding capacity (WHC) were measured following the method described by Fernández‐López *et al*.^18^ and solubility was assessed as described by Cano‐Chauca *et al*.^19^

#### Determination of phenolic compounds

Total phenolic compounds (TPC) were determined by exhaustive extraction, followed by the Folin–Ciocâlteu spectrophotometric method.^20^ The results were expressed as grams of gallic acid equivalents per kg of dry sample (g GAE kg^−1^).

Phenolic compounds were determined and quantified using high‐performance liquid chromatography (HPLC) with the same extract employed for total phenolic content. Chromatographic separation was carried out following the method described by Mallmann *et al*.,^21^ with modifications. An Atlantis C18 column (250 mm × 4.6 mm, 5 μm) was used, in which solvent A was water with 0.1% formic acid and solvent B was acetonitrile with 0.1% formic acid. Elution was conducted with a binary gradient, starting at 5% B and reaching 50.2% B at 46 min. The flow rate was 0.5 mL min^−1^, and the injection volume was 20 μL. A Waters Alliance 2695 HPLC system (Waters Corporation, Milford, MA, USA) coupled to a 2996 diode array detector (DAD) was used for compound identification and quantification. All samples were filtered through a cellulose acetate membrane (0.22 μm) before injection. Analyses were performed in triplicate (*n* = 3). Spectra were recorded between 200 and 600 nm, and the chromatograms were processed at 280 nm. Quantification was based on calibration curves prepared with pyrogallic acid, caffeic acid, dimethoxyhydroxycinnamic acid, 2,5‐dihydroxybenzoic acid, and chlorogenic acid (Table 1).

**Table 1:** Concentration range, *R* ^2^ (coefficient of determination), LOD (limit of detection), and LOQ (limit of quantification) of the phenolic compounds

Phenolic compound	Concentration range (mg L^−1^)	*R* ^2^	LOD (mg L^−1^)	LOQ (mg L^−1^)
Pyrogallic acid	14–140	0.9983	5.59	16.95
Caffeic acid	10–100	0.9992	6.26	18.98
Dimethoxy‐hydroxycinnamic acid	11.1–111	0.9991	6.25	18.94
2,5‐Dihydroxybenzoic acid	14.3–143	0.9971	9.08	27.53
Chlorogenic acid	11.6–116	0.9993	3.52	10.68

#### Determination of betacyanin

Betacyanin extraction and quantification were carried out with adaptations by Sandate‐Flores *et al*.^22^ A 1 g sample was mixed with 10 mL of acidified water (1% v/v acetic acid), homogenized in a tube shaker (Kasvi, Model K40‐1020, São José dos Pinhais, PR, Brazil) for 1 min, and centrifuged at 3500 × *g* for 15 min at 4 °C (Sigma, Model 4K15, Osterode am Harz, Germany). The supernatant was collected, and the extraction was repeated until complete color loss was observed. Betacyanin content was calculated as described by Leong *et al*.:^23^

$$\text{betacyanin concentration}\;\left(\mathrm{BC}\right)=\frac{A\times \mathrm{MW}\times V\times \mathrm{DF}}{\varepsilon \times L\times W}$$

where BC is the betacyanin concentration in mg per 100 g of dry matter; *A* is the absorbance value at the maximum (*λ* max. = 538 nm); *MW* is the molecular weight of betanin (550 g mol^−1^), *V* is the extract volume (mL), *DF* is the dilution factor, *ε* is the molar extinction coefficient of betanin (*ε* = 65 000 L mol^−1^ cm^−1^), *L* is the cuvette path length (cm), and W is the sample weight (g).

#### Color

Color analysis of the residue and flours was performed using a colorimeter (Konica Minolta, Inc., model CR‐400, Tokyo, Japan) based on the CIE Lab* system.

### Evaluation of antifungal capacity

#### Preparation of the residue flour extract

The flour was subjected to an extraction process to obtain bioactive compounds. A total of 5 g of flour was suspended in 50 mL of sterile distilled water (1:10 w/v). The suspension was vortexed at room temperature for 5 min and then sonicated in a water bath sonicator (Unique Group, Indaiatuba, Brazil) for 20 min. Subsequently, the sample was centrifuged at 5000 × *g* for 15 min at 4 °C. The supernatant was collected and filtered through sterile 0.22 μm polyethersulfone (PES) syringe filters (Minisart, Sartorius, Göttingen, Germany) to ensure sterilization of the extract. The extract was stored at 4 °C until use.

#### Fungal strains and inoculum preparation

The fungal strains *Fusarium proliferatum* (clinical isolate) and *Fusarium verticillioides* (corn isolate) were obtained from the Laboratory of Applied Mycology, Federal University of Rio Grande do Sul (UFRGS), Porto Alegre, Brazil. Species identification was carried out through DNA sequencing, and the sequences obtained were compared with those in the *Fusarium* Multilocus Sequence Typing (MLST) database using the Basic Local Alignment Search Tool (BLAST).^24^ The isolates were then cultured on potato dextrose agar (PDA) (Merck, Darmstadt, Germany) plates and incubated for 72 h to obtain fresh mycelial growth for subsequent assays.

#### Mycelial growth inhibition assay

The PDA medium was prepared and sterilized by autoclaving at 121 °C for 15 min. It was then cooled in a water bath to approximately 50 °C. At this stage, the sterile residue flour extract was added to the PDA to obtain final concentrations of 2.5 and 5% (v/v), and the mixtures were homogenized thoroughly.^25^ The PDA containing the extracts (15 mL) was poured into sterile Petri dishes and allowed to solidify. Negative control plates were prepared using sterile PDA without the addition of the extract.

Once the medium had solidified, a 3 mm mycelial plug from each fungal strain (*F. proliferatum* and *F. verticillioides*) was placed in the center of each plate. The plates were incubated at 25 ± 2 °C for 7 days. Mycelial growth was evaluated by measuring colony diameters in two perpendicular directions, and the results were expressed in millimeters. The experiment was conducted in triplicate. The percentage of inhibition (IP%) was calculated at the end of the incubation using the following equation:

$$\mathrm{IP}\;\left(\%\right)=\frac{\left(C-T\right)}{C}\times 100$$

where *C* is the average colony diameter (mm) in the control and *T* is the average colony diameter (mm) in the treatment.

### Application of the flour as a food ingredient

#### Preparation of a bakery product (cake)

A lemon‐flavored cake was prepared using ingredients purchased at a local market in Porto Alegre, RS, Brazil. A control formulation and five formulations with wheat flour substitution levels of 5% to 10% were evaluated in preliminary tests to determine the optimal level. Due to the strong residual flavor observed at substitution levels above 9%, the 9% substitution level was selected for further analysis. The detailed formulations of the control cake and the cake with 9% substitution are presented in Table 2.

**Table 2:** Formulation of the control cake and the cake with partial replacement of wheat flour by flour produced from mixed juice residue

Ingredients	Traditional cake without substitution (%)	Cake with residue flour substitution (%)
Wheat flour (Orquídea)	100	91
Residue flour	0	9
Rolled oat (Nestlé)	18	18
Brown sugar (Da Colônia)	67	67
Egg (Naturovos)	33	33
Sunflower oil (Sinhá)	18	18
Natural yogurt (Nestlé)	83	83
Lemon zest	4	4
Salt (Cisne)	1	1
Baking soda (Kitano)	1	1
Crumbs		
Wheat flour (Orquídea)	5	5
Oatmeal (Nestlé)	12	12
Brown sugar (Da Colônia)	9	9
Butter (Elegê)	9	9
Cinnamon powder (Kitano)	1	1

*Note*: For the calculation of the percentages of both formulations, the wheat flour in the control formulation was used as a basis, representing 100%.

#### Sensory analysis

Cakes prepared with residue flour were evaluated using an attribute acceptance test with 109 untrained panelists. The assessed attributes included appearance, color, aroma, flavor, aftertaste, texture, and overall acceptance, rated on a nine‐point structured hedonic scale (1 = ‘disliked extremely’, 9 = ‘liked extremely’).^26^ Purchase intention was also assessed by asking panelists whether they would buy the product. The results were also expressed as percentages, calculated using the following equation:

$$\text{acceptance index}\;\left(\mathrm{AI}\right)=\frac{M\times 100}{9}$$

where *M* is the mean of the panelists' acceptance scores.

The research was submitted to the ethics committee of the Federal University of Rio Grande do Sul and was approved under the registration number 36 691 414.1.0000.5347.

### Statistical analysis

The experiment was conducted using a completely randomized design. Data were analyzed using analysis of variance (ANOVA), and, when significant differences were detected, means were compared using Tukey's *post hoc* test at a 5% significance level (*P* < 0.05). Statistical analyses were performed using Statistica 13.0 software (StatSoft, Inc., Tulsa, OK, USA).

## RESULTS AND DISCUSSION

### Drying the residue flour

**Figure 1:**
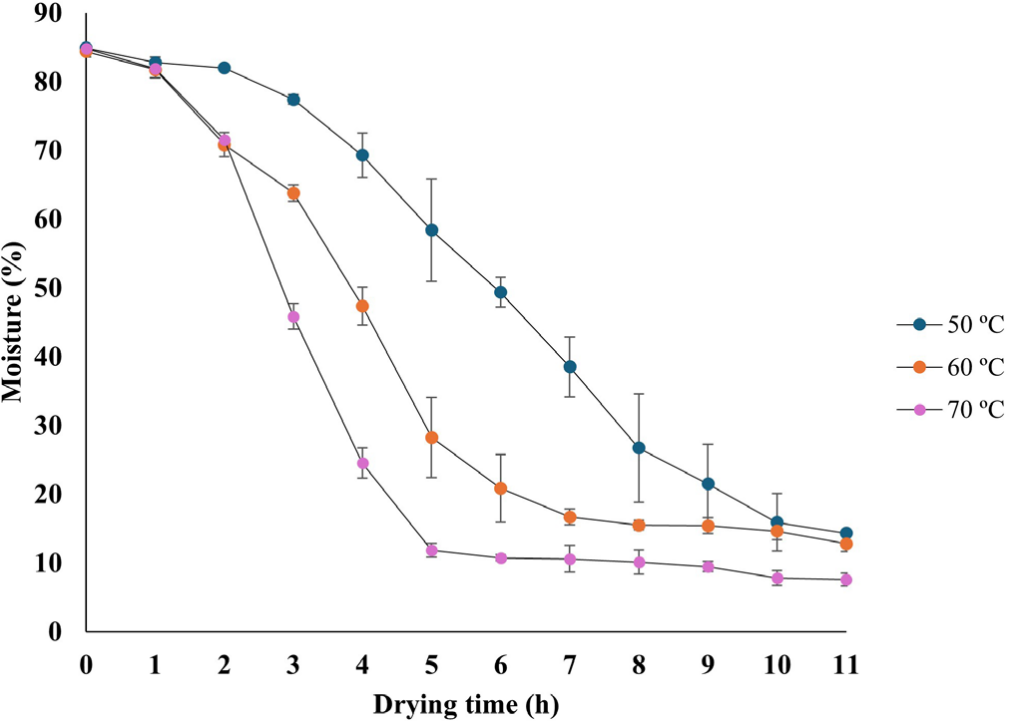
Drying curve of mixed juice residue (apple, beetroot, lemon, and ginger) at 50, 60, and 70 °C. Error bars represent standard deviations (SDs), *n* = 3.

The agro‐industrial residue from mixed juice production had an initial moisture content of 84.5%, typical of residues with high pulp and water content, emphasizing the need for adequate drying to ensure stability and preservation. Figure 1 shows the drying curves at 50, 60, and 70 °C. As expected, drying rate increased with temperature. To reach the target moisture content of 12–15%, 11 h were required at 50 °C, 10 h at 60 °C, and 5 h at 70 °C.

The behavior was expected, as higher drying temperatures increase the thermal gradient and accelerate moisture diffusion from the interior to the surface of the material, reducing drying time significantly.^27^, ^28^ However, elevated temperatures may compromise heat‐sensitive compounds, such as natural pigments and bioactives.

An inverse relationship was observed between drying time and the final concentration of phenolic compounds: prolonged drying at lower temperatures led to greater degradation of bioactive compounds. This indicates that extended thermal exposure was more detrimental to the preservation of the phenolic compounds than a moderate increase in temperature over shorter periods.

The total phenolic content in the fresh residue was 8.42 ± 0.05 g kg^−1^ DM, higher than that observed in the flours obtained after drying. A significant reduction was observed in the dried samples, particularly in the flour dried at 50 °C, which exhibited the lowest content (6.01 ± 0.05 g kg^−1^ DM). Drying at 60 °C and 70 °C resulted in intermediate phenolic concentrations (7.37 ± 0.03 and 7.45 ± 0.08 g kg^−1^ DM, respectively), with no statistically significant difference between them but both were superior and showed a statistically significant difference compared with flour dried at 50 °C. These results indicate that prolonged drying at lower temperatures may be more detrimental to the preservation of phenolic compounds than moderately elevated temperatures applied for shorter periods. This finding is consistent with the observations of Teles *et al*.,^29^ who dried grape pomace at 40, 50, and 60 °C and reported a similar trend, in which lower drying temperatures resulted in lower concentrations of phenolic compounds.

The fresh residue contained 0.0045 ± 0.0007 g kg⁻¹ DM betacyanin, similar to flours dried at 50 °C (0.0043 ± 0.0001 g kg⁻¹ DM) and 60 °C (0.0055 ± 0.0003 g kg⁻¹ DM), with no significant differences among these treatments (*P* > 0.05). In contrast, the flour dried at 70 °C showed a significantly higher concentration (0.0091 ± 0.0005 g kg^−1^ DM), suggesting that the higher temperature, combined with a shorter exposure time, favored pigment release. These results support the hypothesis that thermal processing, within certain limits, can appear to enhance the bioaccessibility of compounds such as betacyanin, possibly due to the breakdown of cellular structures during drying.^15^ These findings are consistent with Kaur *et al*.,^30^ who reported a significant increase in betacyanin concentration in beetroot powder produced after steam blanching, cooling, drying for 8 h at 50 °C, and grinding (14.96 g kg^−1^) compared with fresh beetroot (4.04 g kg^−1^), on a fresh weight basis.

In addition to total phenolics and betacyanins, color parameters were analyzed (Table 3) to determine the optimal drying temperature. The flour dried at 70 °C showed a stronger red hue, and the b* value was lower, reflecting the least yellowing among the three temperatures.

**Table 3:** Color analysis of fresh residues and flours with a particle size of less than 60 mesh, obtained at different drying temperatures

	Fresh residue	Flour dried at 50 °C	Flour dried at 60 °C	Flour dried at 70 °C
L*	21.49 ± 0.12^c^	43.75 ± 0.67^a^	42.09 ± 0.14^a^	37.06 ± 1.08^b^
a*	20.08 ± 0.13^a^	13.23 ± 0.25^c^	12.94 ± 0.31^c^	15.89 ± 0.68^b^
b*	5.18 ± 0.13^c^	11.93 ± 0.29^a^	11.78 ± 0.12^a^	08.45 ± 0.02^b^
*ΔE*	–	24.5 ± 0.61^b^	22.78 ± 0.26^b^	16.47 ± 0.85^a^

*Note*: Results are expressed as the means of three replicates ± standard deviations. Different letters in the same row indicate significant differences (*P* < 0.05) in the Tukey test. *ΔE*: total color difference between the fresh residue and the flours.

The flour obtained by drying at 70 °C exhibited the highest levels of betacyanin and phenolic compounds, and the lowest *ΔE* value, indicating the smallest color variation compared to the fresh residue. Considering that the higher the *ΔE*, the greater the color difference compared to the fresh residue, less color degradation occurred in this flour compared to the parameter values for flours dried at 50 and 60 °C. These results are in agreement with the findings of Costa *et al*.,^15^ who reported that drying beetroot flour at 70 °C led to greater retention of betalains (81.31 ± 0.69 g kg^−1^ DM) and antioxidant activity (71.34 ± 0.94% inhibition) compared with drying at 60 °C (75.05 ± 0.18 g kg^−1^ DM and 65.08 ± 0.09% inhibition) and 80 °C (71.17 ± 0.52 g kg^−1^ DM and 61.14 ± 0.56% inhibition), demonstrating that 70 °C represents an optimal balance between drying efficiency and the preservation of bioactive compounds.

The curve at 70 °C exhibited the most efficient profile in terms of processing time, rapidly reaching the safe moisture range for storage and offering potential energy savings in industrial applications. Based on these results, and considering complementary analyses of color, phenolic compounds, and betacyanin, this condition was selected as the most suitable for flour production from the residue.

In the particle size analysis of the flour, approximately 56% of the particles were retained on a 60 mesh sieve (particles >250μm), 9% corresponded exactly to the 60 mesh fraction, 10% were retained on an 80 mesh sieve (177μm), and 25% passed through a 100 mesh sieve (particles <150μm). To enhance the applicability and functional performance of the flour, it was divided into two fractions: coarse flour, composed of particles retained on a 60 mesh sieve (>250μm), and fine flour, composed of particles passing through a 60 mesh sieve (<250μm).

### Residue flour characterization

Table 4 reports the physicochemical composition and functional properties of the flours. Flour obtained from mixed juice processing residue contains significant amounts of dietary fiber, with a predominance of insoluble fiber. However, soluble fiber is also present in considerable quantities. Soluble and insoluble fibers are both important for human health. Evaluating the particle size fractions revealed that coarse flour has a higher soluble fiber content, whereas fine flour exhibits a higher concentration of insoluble fiber. Despite these differences, the total dietary fiber content of the two fractions is similar, approximately 510 and 490 g kg^−1^, respectively.

**Table 4:** Physicochemical composition and functional properties of flour obtained from apple, beetroot, lemon, and ginger residues

Parameter (DM)	Coarse flour (< 60 mesh)	Fine flour (≥ 60 mesh)
Moisture (%)	12.32 ± 0.47^a^	12.75 ± 0.42^a^
pH	3.77 ± 0.006^a^	3.79 ± 0.02^a^
Ash (g kg^−1^)	40.30 ± 1.49^a^	42.04 ± 1.77^a^
Lipids (g kg^−1^)	18.93 ± 1.33^a^	21.15 ± 1.32^a^
Protein (g kg^−1^)	66.21 ± 4.76^a^	70.61 ± 3.72^a^
Soluble fiber (g kg^−1^)	167.63 ± 0.57^a^	105.85 ± 9.41^b^
Insoluble fiber (g kg^−1^)	343.95 ± 20.86^b^	382.58 ± 2.61^a^
Total fiber (g kg^−1^)	511.58 ± 21.44^a^	488.45 ± 6.80^a^
Carbohydrates (g kg^−1^)^a^	868.90 ± 6.86^a^	860.05 ± 4.80^a^
Aw	0.405 ± 0.006^b^	0.480 ± 0.003^a^
Solubility (g/100 mL)	392.20 ± 11.90^a^	406.50 ± 2.20^a^
WHC (g water g^−1^ flour)	11.36 ± 0.60^a^	10.61 ± 0.25^a^
OHC (g oil g^−1^ flour)	2.63 ± 0.09^b^	2.92 ± 0.02^a^

^a^ Carbohydrate content was estimated by difference. Aw water activity; WHC water holding capacity; OHC oil holding capacity. Results are expressed as the means of three replicates ± standard deviations. Different letters in the same row indicate significant differences (*P* < 0.05) in the Tukey test.

These components confer several physiological benefits. Soluble fiber, which is more abundant in the coarse flour, is well recognized for modulating intestinal health,^31^ improving glycemic control^32^, ^33^ and reducing cholesterol levels.^34^, ^35^ Insoluble fiber, which predominates in the fine flour, increases fecal bulk and regulates intestinal transit.^36^, ^37^

Dietary fibers are valued not only for their health benefits, but also for their applications in food products. For example, enzyme‐modified insoluble ginger fiber can be used as an additive in bakery products, meats, and beverages to improve WHC and to act as a stabilizer.^38^ Given the relevance of dietary fibers, the flour produced from mixed juice residue, regardless of particle size, represents a promising source of functional fibers to contribute significantly to a healthy diet.

Both flours can therefore be considered valuable sources of dietary fiber, given their high fiber content. They also show low lipid content and relatively low ash and protein levels, consistent with the typical composition of fruits and vegetables. Similar results were reported by Coimbra *et al*.^39^ for beetroot peel flour, with 307.0 ± 18.1 g kg^−1^ of total fiber, 15.4 ± 2.60 g kg^−1^ of lipids, and 64.2 ± 1.30 g kg^−1^ of ash. Santos *et al*.^40^ reported comparable values for a breakfast cereal composed of apple residue flour and wheat bran (70% apple:30% wheat bran), with 411.00 ± 11.00 g kg^−1^ of total fiber, 29.00 ± 0.00 g kg^−1^ of ash, and 67.00 ± 0.00 g kg^−1^ of protein.

The Aw values differed significantly, 0.405 ± 0.001 being observed for the coarse particle sizes and 0.480 ± 0.003 for the fine particle sizes. These values are comparable with those reported by Crizel *et al*.^16^ for orange flour produced from peel, pomace, and seeds (0.430 ± 0.04). Low Aw is essential to inhibit microbial growth and ensure the stability and shelf life of the flour during storage, preventing microbial deterioration, spoilage, and undesirable physicochemical changes. Carter *et al*.^41^ also pointed out that the higher the water activity in wheat flour, the higher was the risk of mold and rancidity, particularly at Aw above 0.7.

Regarding the functional properties of the flours, no significant differences were observed between the two particle sizes in terms of solubility and WHC. The flour presented high hydration capacity (11.36 ± 0.60 g water g^−1^ flour for coarse flour and 10.61 ± 0.25 g water g^−1^ flour for fine flour) compared, for example, with Wang *et al*.,^42^ which showed a WHC of 1.18 ± 0.03 g water g^−1^ flour in flour enriched with pea protein without any prior treatment.

The large hydration capacity is important both for the flour's use as a thickener and in the preparation of breads and cakes, promoting dough viscosity and structure while ensuring adequate moisture and texture. Filipčev *et al*.^43^ reported that incorporating psyllium, which increases WHC, improved gluten‐free bread texture, yielding a softer and more elastic crumb. The OHC can also help improve food texture and flavor,^44^, ^45^ and a statistically significant difference was observed for this parameter. In practical applications, however, such as in the cake tested, this difference is negligible, with values of 2.92 ± 0.02 for the fine flour and 2.63 ± 0.09 for the coarse flour.

The identification and quantification of phenolic compounds in the two flour fractions obtained from mixed juice residue revealed notable differences in their bioactive compound profile (Table 5). Ten phenolic compounds were identified by HPLC, with important variations between particle sizes. Fine flour showed higher concentrations of compounds such as pyrogallic acid, *m*‐coumaric acid, 2,5‐dihydroxybenzoic acid, catechin, and chlorogenic acid. The coarse flour exhibited a higher content of *p*‐coumaric acid, whereas other compounds, such as gallic acid and protocatechuic acid, did not show a significant difference between the fractions.

**Table 5:** Identification and quantification of phenolic compounds of flour obtained from apple, beetroot, lemon, and ginger residues

Identified phenolic compounds (g kg^−1^)	Coarse flour (< 60 mesh)	Fine flour (≥ 60 mesh)
Pyrogallic acid	1.59 ± 0.04^b^	1.73 ± 0.02^a^
*m*‐Coumaric acid	14.83 ± 0.25^b^	17.82 ± 0.71^a^
*p*‐Coumaric acid	0.88 ± 0.20^a^	0.26 ± 0.01^b^
Caffeic acid	0.0066 ± 0.0001^a^	0.0096 ± 0.0027^a^
Dimethoxy‐hydroxycinnamic acid	0.05 ± 0.01^b^	0.07 ± 0.01^a^
2,5‐Dihydroxybenzoic acid	5.70 ± 0.13^a^	7.30 ± 0.99^a^
Catechin	1.59 ± 0.06^b^	2.43 ± 0.38^a^
Chlorogenic acid	0.12 ± 0.01^b^	0.15 ± 0.01^a^
Gallic acid	2.98 ± 0.71^a^	2.20 ± 0.55^a^
Protocatechuic acid	2.25 ± 0.02^a^	2.36 ± 0.12^a^
Total	**30.00 ± 0.79**	**34.33 ± 1.40**

*Note*: Results are expressed as the means of three replicates ± standard deviations. Values are expressed for dry matter (DM). Different letters in the same row indicate significant differences (*P* < 0.05) in the Tukey test.

The fine flour contained approximately 34.33 ± 1.40 g kg^−1^ of phenolic compounds, compared with 30.00 ± 0.79 g kg⁻¹ in the coarse fraction. This difference may result from the smaller particle size of the fine fraction, which likely promotes greater disruption of cellular structures during milling, increasing particle surface area and facilitating access to phenolic compounds bound to the plant matrix.^46^ Similar observations were reported by Tian *et al*.,^47^ who found higher concentrations of phenolic acids in wheat flours with smaller particle sizes. From a biological standpoint, higher phenolic concentrations may indicate greater antioxidant potential, as demonstrated by Yari *et al*.,^48^ who reported a strong positive correlation between phenolic compounds and antioxidant activity in *Physalis* extracts. However, biological effects were not evaluated directly in the present study. The observed differences should therefore be interpreted as indicative of functional potential rather than confirmed bioactivity.

Filip *et al*.^49^ analyzed freeze‐dried residues of golden apples, red apples, carrots, celery, beetroot, and pink‐skinned potatoes. Among the phenolic compounds that they identified, flour, catechin and gallic acid were detected in all residues whereas *p*‐coumaric acid was not detected in the beetroot residue. It was observed that, among these three phenolic compounds, two were present in higher quantities in both flour fractions: gallic acid, which showed a concentration approximately 4.9 times higher than that found in the celery residue (0.604 g kg^−1^ DM), and *p*‐coumaric acid, with concentrations around 5.7 times greater than that in the golden apple residue (0.15 g kg^−1^ DM) in the coarse flour fraction. In contrast, catechin exhibited a concentration approximately 5.8 times lower than that found in the celery residue (14.06 g kg^−1^ DM) when compared with the fine flour fraction of the residue flour.

Flours from mixed juice residue represent significant sources of phenolic compounds, exhibiting higher concentrations than those reported in other studies evaluating plant residues. The exception was catechin in the Filip *et al*.^49^ study, which was present in lower amounts in the mixed juice flours than the celery, beetroot, and carrot residues.

### Antifungal activity

**Figure 2:**
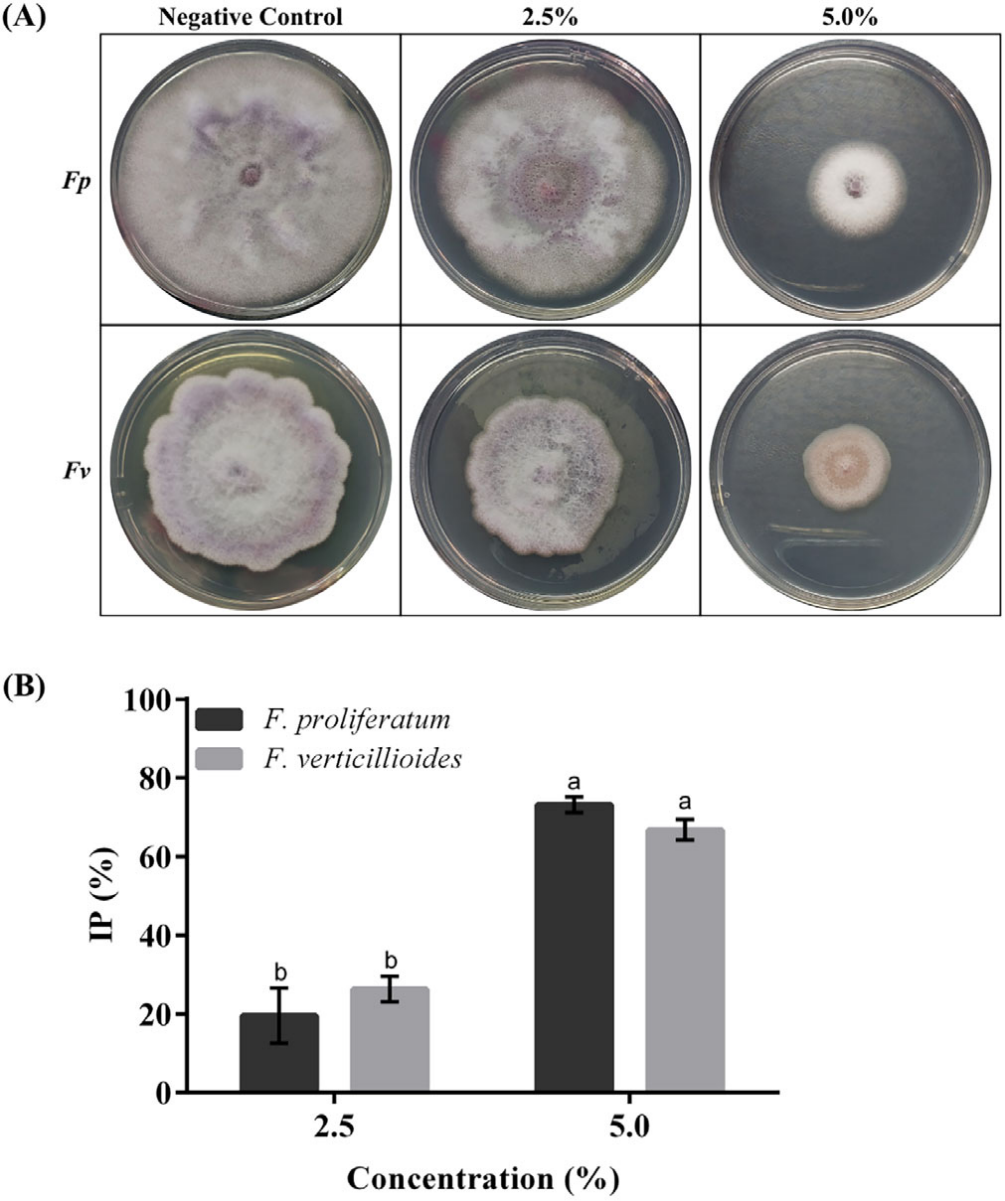
(A) Inhibition of mycelial growth of *Fusarium proliferatum* (*Fp*) and *F. verticillioides* (*Fv*) at different concentrations of residue flour extract (2.5 and 5%, v/v). (B) Percentage of inhibition (IP, %) calculated from the reduction of colony diameters. Different lower case letters indicate statistically significant differences (*P* < 0.05) from a two‐way ANOVA followed by a *post‐hoc* test.

The antifungal activity of the residual flour was evaluated at two concentrations (2.5% and 5%) against two *Fusarium* species (Fig. 2). Activity was strongly concentration dependent, with a significant increase in inhibition observed from 2.5% to 5.0% (*P* < 0.0001). At 5%, the mean inhibition percentages were 73.18 ± 2.01% for *F. proliferatum* and 68.79 ± 2.62% for *F. verticillioides*. Two‐way ANOVA revealed a significant interaction between concentration and species (*P* = 0.0266); Tukey's *post‐hoc* test subsequently indicated no significant difference in inhibition between the two species at the same concentration (*P* > 0.05). Overall, a 5% concentration was significantly more effective than a 2.5% for both *Fusarium* species.

The enhanced inhibitory effect observed at the higher concentration highlights the dose–response relationship of natural extracts, reflecting the direct influence of bioactive compounds on antifungal efficacy.^25^, ^50^ This fungistatic effect can be attributed to the rich phytochemical composition of the blended residues, which include high levels of total phenolic compounds and specific bioactive molecules such as catechin, chlorogenic acid, and gallic acid.

Chlorogenic acid and catechin further support antifungal activity by damaging membranes and interfering with key enzymatic processes.^50^ Compounds derived from ginger, lemon, apple, and beetroot likely contribute to this activity. Gingerols and other phenolics from ginger have been reported to disrupt mycelial morphology and increase cell membrane permeability in *Fusarium* species,^51^ and lipophilic essential oils and terpenes in lemon residues, including limonene, can destabilize fungal cell membranes, causing leakage of cellular contents and fungal death.^52^, ^53^ Beet residues may also contribute to the overall antifungal activity, as hydroalcoholic extracts of *Beta vulgaris* leaves have been reported to exhibit antifungal effects against *Candida albicans*, due to their rich phenolic and saponin content.^54^

Although small differences between species were noted, the extract showed comparable inhibition against both *Fusarium* species, suggesting that bioactive compounds act on conserved physiological pathways.^50^, ^51^ The strong efficacy observed, particularly at 5.0% concentration, highlights the value of using complex food‐industry byproducts for the sustainable development of natural preservatives, offering both environmental benefits and effective antifungal protection.^54^, ^55^ These results should be interpreted as a preliminary screening and do not reflect the performance of the flour directly when incorporated into baked food products. Additional studies are necessary to determine whether this activity is preserved after baking processes.

### Application of the residue flour

Although the fine flour exhibited a higher content of phenolic compounds, which contribute to its antioxidant and functional properties, the coarse flour was selected for cake formulation due to its more favorable sensory profile. Phenolic compounds often impart bitter, astringent, or residual flavors that may reduce consumer acceptance, and the higher concentration of these compounds in the fine flour could intensify these notes. In contrast, the coarse flour, despite its lower phenolic content, provides a more balanced and acceptable flavor in the cake, ensuring better palatability without compromising nutritional quality. This balance between functional value and sensory acceptability is fundamental in developing food ingredients from agro‐industrial residues, particularly when aiming to enhance health benefits while maintaining product appeal.

Mixed juice residue flour was used to partially replace wheat flour in the preparation of lemon‐flavored cake, to increase its dietary fiber content. The control cake contained 28.7 g kg^−1^ of fiber, whereas the formulation, in which 9% of the wheat flour was substituted with mixed juice residue, reached 40.28 g kg^−1^ of fiber, representing an approximately 40% increase in total fiber. Representative images of the control formulated cakes are provided in the Supporting Information; however, no visual differences were observed between them.

The substitution or incorporation of fruit or residue flours is used widely to improve the nutritional composition of food products. Several studies illustrate the broad application of different flours in various formulations. For instance, Jofre *et al*.^56^ used freeze‐dried grape pomace flour in the production of gluten‐containing cookies, obtaining products with higher protein and fiber content. Santos *et al*.^40^ incorporated apple residue flour together with wheat or rice bran in the production of extruded breakfast cereals to develop a high‐fiber product, achieving a total fiber content of 260 g kg^−1^. Özdemir *et al*.^57^ used carob molasses pulp flour as to replace part of the wheat flour in the production of cakes, yielding a product with a higher quantity of insoluble fiber, polyphenols, and antioxidant content.

Table 6 reports the results of the sensory analysis. All acceptance scores were above 70%, indicating a high level of consumer approval for the lemon‐flavored cake enriched with mixed juice residue flour. Regarding purchase intention, 70% of respondents indicate that they would buy the product, which highlights its strong market potential and consumer acceptability.

**Table 6:** Results of the sensory analysis of lemon‐flavored cake enriched with residue flour obtained from mixed juice processing

Attributes	Assigned score	Acceptance index (%)
Appearance	6.71 ± 1.49	74.5
Color	6.59 ± 1.69	73.2
Aroma	7.83 ± 1.21	87.1
Flavor	7.47 ± 1.55	83.0
Aftertaste	6.59 ± 2.04	73.2
Texture	7.28 ± 1.85	80.8
Overall acceptance	7.26 ± 1.26	80.6

*Note*: Scores are presented as means ± standard deviations from 109 panelists, where 1 = disliked very much, 2 = disliked a lot, 3 = disliked moderately, 4 = disliked slightly, 5 = neither liked nor disliked, 6 = liked slightly, 7 = liked moderately, 8 = liked a lot, and 9 = liked extremely.

The cake achieved an acceptance index of 80.6%, a value considered positive and above the minimum threshold typically regarded as an indication of good acceptability for food products. Among the evaluated attributes, aroma and flavor received the highest scores, indicating that flour incorporation did not compromise the cake's olfactory characteristics and was even perceived as one of its strengths. Palatability was not negatively affected, despite the potential for residual flavors from bioactive compounds. The aftertaste presented an acceptance index of 73.2%, indicating that, although perceptible, it did not significantly impact the overall acceptance of the product. These results support the choice of the coarser particle size flour for the formulation, as its lower phenolic compound content likely contributed to the reduction of possible bitter or astringent notes.

The appearance and color attributes had acceptance indices of 74.5 and 73.2%, respectively, indicating good acceptance of the cake's overall visual appeal. These scores were slightly lower than those for other attributes, possibly because the cake resembled whole‐grain products, influenced by the brown color from brown sugar and the grainier texture contributed by fiber and ingredients such as oats. The texture showed an acceptance index of 80.6%, demonstrating that fiber incorporation did not adversely affect the mouthfeel of the product, preserving a pleasant sensory experience.

The previously cited studies also reported sensory analysis results. For instance, Jofre *et al*.^56^ reported that the cookie with the highest overall acceptance scored 7.2 on a 9‐point hedonic scale. In contrast, Mateus *et al*.^58^ found that muffins formulated with lemon pomace flour showed lower acceptability due to noticeable bitterness in comparison with other formulations; however, no specific numerical values were provided for direct comparison with the sensory results of the present study. Özdemir *et al*.^57^ reported overall acceptance values ranging from 78% to 90% for cakes in which wheat flour was partially replaced with carob molasses pulp flour. Like the present findings, these results indicate that food waste can be incorporated successfully into products such as cakes, contributing to product enrichment and the sustainable use of by‐products.

In the current study, even with the addition of mixed juice residue, which can impart a slightly bitter aftertaste due to lemon residue, no consumer rejection was observed. The results demonstrated the feasibility of incorporating mixed juice residue flour into bakery products, contributing to nutritional enrichment while promoting the valorization of agro‐industrial by‐products and supporting a more sustainable food production model.

## CONCLUSIONS

The present study demonstrated that residues generated from mixed juice production, composed of apple, beetroot, lemon, and ginger, can be transformed successfully into a functional flour with high nutritional and technological potential. The highest drying temperature for the shortest amount of time was identified as the optimal condition among the conditions tested, providing the preservation of bioactive compounds and color attributes. Both fractions of the flour exhibited significant dietary fiber content, with the coarse fraction richer in soluble fiber and the fine fraction in insoluble fiber, while maintaining favorable physicochemical and functional properties. The flour also demonstrated antifungal activity against *Fusarium* species.

High‐performance liquid chromatography analysis identified ten phenolic compounds, and particle size influenced their availability, with finer particles facilitating greater extraction. Application of the coarse flour in a lemon‐flavored cake resulted in increased dietary fiber content, high sensory acceptance, and satisfactory purchase intention.

Overall, the study highlights the potential of valorizing agro‐industrial residues to develop functional ingredients that combine nutritional enhancement, technological applicability, and consumer acceptance, contributing to sustainability in the food industry and promoting the full utilization of food resources. These findings align with the goals of the UN (United Nations) 2030 Agenda for Sustainable Development. However, further research is needed to assess the retention of phenolic compounds following food processing, along with their application in other food products.

## CONFLICTS OF INTEREST

The authors declare no conflicts of interest.

## Supporting information


**Figure S1.** Visual appearance of the control cake (without residue flour) and the cake formulated with residue flour substitution.

## Data Availability

The data that support the findings of this study are available from the corresponding author upon reasonable request.
