# Supplementary figures and images for: Physicochemical characterization and phenolic compound content of flour from blended juice residues
Source: J Sci Food Agric. 2026 Feb 19;106(7):4331–40. doi: 10.1002/jsfa.70523 (PMC13067078; doi:10.1002/jsfa.70523)

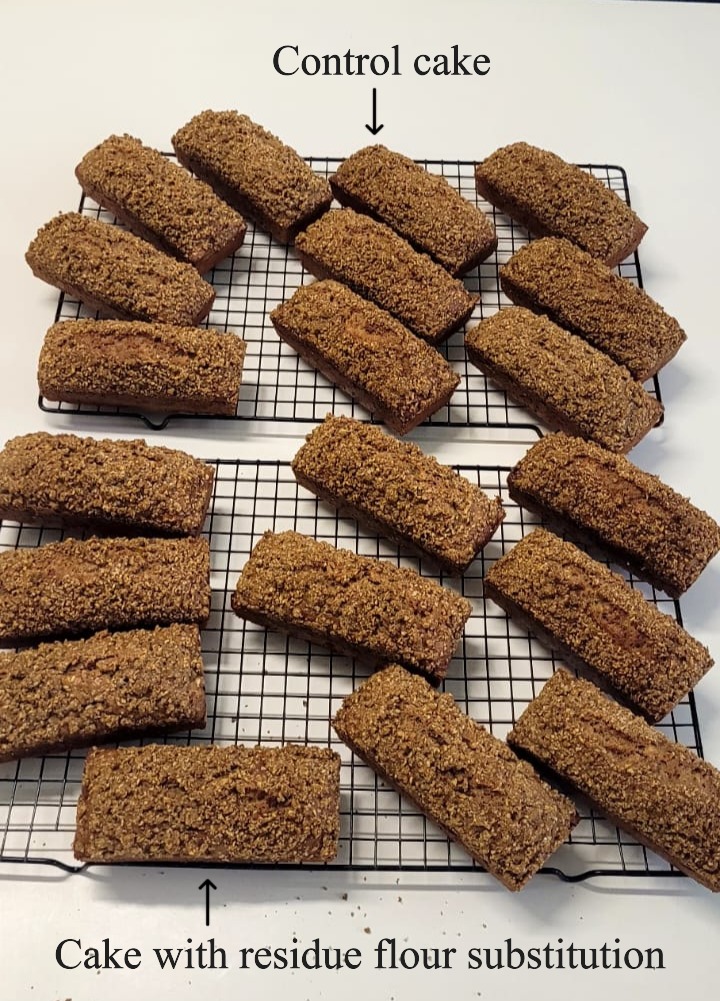

Supplement: Supplementary file 1 — Figure S1. Visual appearance of the control cake (without residue flour) and the cake formulated with residue flour substitution. [file JSFA-106-4331-s001.jpg]
